# The challenges of implementing infection prevention and antimicrobial stewardship programs in resource-constrained settings

**DOI:** 10.1017/ash.2024.35

**Published:** 2024-04-16

**Authors:** Salma Abbas

**Affiliations:** Department of Internal Medicine, Shaukat Khanum Memorial Cancer Hospital and Research Center, Lahore, Punjab, Pakistan

## Abstract

The burden of healthcare-associated infections (HAIs) and antimicrobial resistance (AMR) is disproportionately high in low and middle-income countries. Barriers to implementing effective antimicrobial stewardship and infection prevention programs include the lack of a structural framework, consensus guidelines, educational opportunities, trained personnel, funding, and access to resources such as manpower, information technology, and diagnostics. Socioeconomic instability with supply chain interruptions, poor skilled staff retention, absence of mandates, and inadequate support to enforce existing policies further aggravates the situation. Failure to implement measures to tackle AMR and HAIs effectively will result in repercussions globally.

Antimicrobial resistance (AMR) and healthcare-associated infections (HAIs) such as catheter-related bloodstream infections (CRBSIs), catheter-associated urinary tract infections (CAUTIs), ventilator-associated pneumonias (VAPs), and surgical site infections represent major healthcare challenges globally, with the burden being disproportionately higher in low and middle-income countries (LMICs).^1–3^ These lead to prolonged hospital lengths of stay (LOS), adversely impact patient outcomes, and inflate healthcare bills.^3^ Inappropriate use of antibiotics leads to adverse events associated with antibiotics, higher healthcare costs, emergence of multidrug-resistant organisms, and infections such as *Clostridioides difficile*.^4,5^ Programs designed to target infection prevention and control (IPC) and antimicrobial stewardship (AS) are inter-dependent and share several similarities including process and outcome metrics and resources.^6^ Incorporation of these programs at healthcare facilities can reduce HAIs, help combat AMR, and improve health outcomes.^6^

Reporting of AMR and HAI data is mandatory in many developed countries.^7^ In response to the 2017 WHO report on the global priority list of antibiotic-resistant bacteria, 21 of 32 countries in the European Union and European Free Trade Association member states implemented mandatory surveys for multidrug-resistant organisms, and 15 provided structural framework for surveillance.^7^ On the contrary, mechanisms for reporting are lacking in LMICs and high-quality data are sporadic and limited to institutions and hospitals.^8^ The WHO instituted the global action plan (GAP) to combat AMR in 2015 calling all countries to devise national action plans (NAPs) to combat AMR. Agencies such as the Food and Agriculture Organization of the United Nations and the World Organization for Animal Health have also adopted the GAP. In 2016, the United Nations General Assembly released a declaration acknowledging that humans, animals, plants, and the environment are inter-linked and, a ‘One Health’ approach targeting multiple sectors such as healthcare, industry, agriculture, and livestock, all of which contribute the threat of AMR, must be adopted.^9–11^ Following this, NAPs have been launched in 33 countries in Africa, 14 countries in the Americas, 20 countries in the Eastern Mediterranean, 36 countries in Europe, 11 countries in Southeast Asia, and 22 in the Western Pacific.^12^ These action plans address key issues associated with AMR and outline strategies to counter these by addressing knowledge gaps, enhancing surveillance and research, optimizing the use of antibiotics, reducing HAIs, and mobilizing funding to sustain AS activities.^12,13^ Most LMICs collecting AS data report to the WHO Global AMR Surveillance System (GLASS).^12^ These countries follow individual timelines and reporting intervals. The scope of these programs only allows for a broad-stroke characterization rather than granular assessment as data is collected from a few institutions and with limited geographical representation.

In Europe, HAIs are estimated to prolong hospital LOS by 16 million days, with 37 000 attributable deaths annually. The annual direct healthcare costs are estimated at approximately € 7 billion.^14^ According to a World Health Organization (WHO) report, crude excess mortality attributable to device-associated HAIs in 173 ICUs from 25 countries in Latin America, Asia, Africa, and Europe was estimated at 18.5%, 23.6%, and 29.3% for CAUTI, CRBSI, and VAP, respectively with attributable excess hospital LOS between 5 and 29.5 days.^14^ Another study estimating the global burden of AMR through statistical modeling reported that 4·95 million deaths were associated with bacterial AMR, with 1·27 million (95% uncertainty intervals 0·911–1·71) deaths attributable to bacterial AMR in 2019, including 0.86 million deaths in Africa.^15^ This underscores the importance of robust measures in LMICs to ensure reliable surveillance and timely implementation of necessary measures to improve health outcomes.

## The challenges of implementing antimicrobial stewardship programs (ASPs) in resource-constrained settings

### Lack of a structural framework and funding for AS activities

A total of 136 countries have launched NAPs to combat AMR globally. However, successful implementation of ASPs is limited by several factors.^12,16^ According to the WHO Global Database for Tracking AMR Country Self-Assessment Survey 2023, multi-sectoral coordination mechanisms on AMR exist in most LMICs. However, functional working groups, funding for surveillance activities, and accountability are lacking.^16^ This may be attributed to the heavy reliance of LMICs on external funding sources such as the Fleming Fund, the WHO, and the U.S Centers for Disease Prevention and Control (CDC). As most of these are short-term, sustainability remains a key challenge.^17^ Moreover, while the gravity of AMR is acknowledged at the government level, the lack of a formalized reporting and surveillance mechanism, lack of consensus guidelines, and failure to mandate AS activities prevent the effective downstream dissemination of information and implementation of ASPs at healthcare facilities.^12,16^

### Shortage of qualified professionals and limited access to resources such as diagnostics and information technology

AS activities are limited by a shortage of trained personnel, including pharmacists and physicians to lead AS initiatives at healthcare facilities.^18,19^ Most hospitals lack diagnostic facilities and advanced testing such as rapid molecular diagnostics to support AS activities are largely unavailable.^11^ Injudicious prescription of antibiotics is rampant, with a high proportion of antibiotics prescribed empirically and without a clear indication.^11^ A handful of hospitals with trained healthcare professionals and availability of resources such as pharmacy and microbiology support, access to information technology, and human resource provisions, may support institutional AS initiatives but the scope of these activities remains very limited (Table 1). Consequently, without granular data on AMR trends, existent policies to combat AMR remain myopic and are unable to identify and address critical gaps.

**Table 1.** Challenges in implementing infection control and antimicrobial stewardship programs in resource-constrained settings

Element	IPC and AS challenges
Leadership support	• Inadequate leadership support
Structure	• Variably structured programs, where present
Accountability	• No mechanism to ensure accountability
Resource allocation for supplies	• Limited resource allocation for supplies such as hand sanitizers, disinfectants, PPE • Poor access to diagnostics including molecular tests for early diagnosis of infections
Human resource and expertise	• Shortage of trained professionals and training programs in IPC and AS • Failure to retain trained professionals due to socioeconomic instability
IT support	• Limited IT support
Data Monitoring and reporting	• Absence of central reporting structure • No local benchmarks for comparison across similar facilities
Sociopolitical factors	• Supply chain disruptions due to frequent policy changes and socioeconomic instability • Compartmentalization of health from economic and agricultural sectors
Role of regulatory bodies	• Lack of consensus guidelines, regulation, and enforcement of recommended practices • No mandates to ensure the implementation of IPC and AS programs

Note. IPC, infection prevention and control; AS, antimicrobial stewardship; PPE, personal protective equipment; IT, information technology.

### Self-medication and access to antibiotics over-the-counter

The culture of self-medication and unhindered access to antibiotics over-the-counter fuels AMR.^20^ A cross-sectional study from Pakistan assessing the sociodemographic factors associated with antibiotic self-medication revealed that approximately 70% of survey participants had used antibiotics over the past year and 30% without consultation with a healthcare professional.^20^ Similarly, a survey of 5 countries in Africa revealed that antibiotics are among the most frequently prescribed medications, with 90% of individuals with acute illness seeking care and 36% being prescribed antibiotics. About 30% of these patients received antibiotics without prescriptions and 25% received antibiotics from informal dispensers.^21^ The issue is further aggravated in countries such as Afghanistan where international borders are poorly secured, smuggling is rampant, and access to medications, including antibiotics, is not regulated.^13^

### Compartmentalization of health from agricultural and economic sectors

The healthcare sector is, naturally, the initial focus of AMR prevention efforts. However, the concept of One Health is still in its infancy in most LMICs and healthcare represents the sole focus of AMR prevention activities. The compartmentalization of various sectors and failure to recognize the contribution of pharmaceutical and other chemical industries, livestock businesses and agriculture sectors, and inadequate effluent management in municipal systems further promote AMR.^10^ According to an estimate, about 60% of all human pathogens are zoonotic and the increasing human-animal interaction poses a high risk for acquiring these infections.^22^ Antibiotics can be easily purchased over the counter in LMICs and are used for infection prophylaxis, growth promotion, and as food additives in the agriculture and livestock industries. Additionally, improper waste disposal, poor hygiene and sanitation, overcrowding of animals, and failure to use human protective gear while handling animals are key contributors to the surge of AMR among animals.^22^

## The challenges of implementing infection prevention and control (IPC) programs in resource-constrained settings

### Absence of mandates and poor organizational structure

Data on IPC activities in LMICs are limited. This is highly concerning given the disproportionately high burden of HAIs in low LMICs.^2^ Like AS, IPC activities are not mandated, and a structural framework to ensure the implementation of these programs is absent. Additionally, funding for IPC is variable, structured training is limited and accountability is lacking.^8,23^

### Shortage of trained professionals

The impact of IPC programs is dampened by a lack of trained professionals, including infectious diseases physicians, microbiologists, and infection prevention nurses (IP), limiting IPC programs to a few hospitals and preventing large-scale implementation of IPC activities.^18^ Hospitals taking IPC initiatives struggle with staff retention due to sociopolitical instability. A report published by the Bureau of Emigration and Overseas Employment, Pakistan, revealed that an alarming number of skilled and highly skilled professionals immigrate to developed countries, including about 1000 doctors per year.^24^ The lack of trained professionals is evident from a recent survey of 18 hospitals with IPC programs, where only 3 IPC program chairs reported having received formal training and 25% failed to meet the IP staffing criteria as recommended by the WHO.^8^

### Scarce resources, supply constraints, and unavailability of facilities such as isolation rooms and engineering controls

Other major barriers to the implementation of IPC programs include a lack of support from hospital leadership, shortages of supplies such as PPE, hand sanitizers and disinfectants, lack of consensus guidelines, opposition from hospital staff regarding recommended practices, lack of engineering controls and negative pressure rooms, lack of adequate space and isolation rooms, and the absence of local benchmarks for comparison of IPC data across facilities (Table 1).^8,25^

### Lack of access to health insurance

Another key consideration is prohibitively high health insurance costs in LMICs, with approximately 40%–60% of healthcare expenditure being out-of-pocket.^26,27^ Consequently, HAIs are not tied to reimbursement for healthcare facilities and carry no repercussions.

### Experience from LMICs

While AS and IPC initiatives are in their infancy in most LMICs, some countries including Tanzania, Cameroon, Ethiopia, and Mongolia have progressed from initial NAPs to the next tier of AS activities.^12^ In Tanzania, the first AMR NAP (2017–2022) primarily focused on the human sector and was limited to the national and ministerial levels with intra and inter-sectoral variation in the achievement of objectives. To address these gaps, the follow up plan for 2023-2028 outlines strategies for successful implementation at the regional, district and council levels with engagement of the animal, plant and environment sectors and includes strengthening of coordination, collaboration and governance as a strategic objective.^28^ Similarly, although an AMR governance structure and a One Health steering committee were established in Ethiopia in response to the initial AMR NAP, poor coordination and a lack of collaboration among various stakeholders, scarce resources, shortage of trained personnel, limited surveillance capacity, poor-quality data to inform policies, failure to implement evidence-based practices and poor regulation of antimicrobial use limited the scope of AS activities.^11^ The third edition of Ethiopia’s NAP underscores the importance of sustained action to prevent, control and treat infections, enhanced inter-sectoral collaboration, and incorporation of a One Health approach to successfully combat AMR.^11^ In India, the initial NAP recognized AS as a key strategy to address AMR, however, a national AS strategy was not devised. The AS initiative was launched by the country’s medical council in 2017 with 20 participating hospitals and a meager budget of US $15000 annually.^19^ Despite the limited scope, this initiative has resulted in the establishment of ASPs at 20 tertiary care hospitals and serves as a pilot for the large-scale implementation of AS activities in LMICs.^19^ A phased approach has also been introduced in India in collaboration with the CDC to enhance HAI surveillance at healthcare facilities.^29^

## Conclusion

The current state of affairs in LMICs represents a Swiss cheese model, where deficiencies at multiple levels align to skew the overall burden of HAIs and AMR towards these countries. There is a dire need to prioritize HAIs and AMR as leading health issues and urgently intensify prevention efforts. Setting achievable short and long-term goals with strict adherence to timelines, judicious allocation of resources and graduated implementation of effective measures, starting with low-cost but high-impact interventions such as hand hygiene is key to the success of these programs. The implementation of these activities at a large scale, with individual healthcare facilities feeding into a central body will allow for enhanced surveillance and identification of focus areas. Failure to do so will lead to deleterious consequences, with global ramifications. Moreover, political engagement and a concerted, One Health approach, is essential to ensure that the health and economic sectors work in tandem to achieve a common goal of improving health globally. Sustained funding by international agencies will further the mission and ensure the success of these efforts in LMICs.
